# Targeting ARID1A-mutant colorectal cancer: depletion of ARID1B increases radiosensitivity and modulates DNA damage response

**DOI:** 10.1038/s41598-019-54757-z

**Published:** 2019-12-03

**Authors:** B. Niedermaier, A. Sak, E. Zernickel, Shan Xu, M. Groneberg, M. Stuschke

**Affiliations:** Department of Radiotherapy, University of Duisburg-Essen, University Hospital, Essen, Germany

**Keywords:** Radiotherapy, Cancer epigenetics, Colorectal cancer

## Abstract

The SWI/SNF chromatin remodeling complex has been found mutated in a wide range of human cancers, causing alterations in gene expression patterns, proliferation and DNA damage response that have been linked to poor clinical prognosis. Here, we investigated weather knockdown of ARID1B, one of two mutually exclusive subunits within the SWI/SNF complex, can sensitize colorectal cancer cell lines mutated in the other subunit, ARID1A, to ionizing radiation (IR). ARID1A-mutated colorectal cancer (CRC) cell lines are selectively sensitized to IR after siRNA mediated ARID1B depletion, as measured by clonogenic survival. This is characterized by a decrease in the surviving cell fraction to 87.3% ± 2.1%, 86.0% ± 1.1% and 77.2% ± 1.5% per 1 Gy compared with control siRNA exposed cells in the dose range of 0–6 Gy for the LS180, RKO and SW48 lines, respectively (p < 0.0001, F-test). The magnitude of this dose modifying effect was significantly larger in ARID1A mutated than in non-mutated cell lines (Spearman rank correlation rs = 0.88, p = 0.02). Furthermore, initial formation of RAD51 foci at 4 h after IR, as a measure for homologous recombination repair, was significantly reduced in ARID1A-mutant CRC cell lines but not in the majority of wildtype lines nor in fibroblasts. These findings open up perspectives for targeting ARID1B in combination with radiotherapy to improve outcomes of patients with ARID1A-mutant CRC.

## Introduction

The multiprotein chromatin remodeling complex SWI/SNF plays a key role in the dynamic regulation of gene expression, and mutations of its subunits have repeatedly been linked to human malignancies. Literature findings suggest a wide-spread role of alterations in the expression of SWI/SNF subunit genes in human cancer susceptibility and patient survival times^1^. ARID1A and ARID1B are two mutually exclusive regulatory subunits of the SWI/SNF complex. Notably, mutations of ARID1A were found in several cancers. Wei and colleagues described loss of ARID1A in colorectal cancer being associated with late TNM stage, poor pathological classification and distant metastasis, and concluded that patients with ARID1A-mutated cancers may benefit from therapy targeting chromatin modifying enzymes^2^. Recent studies identified ARID1B as a potentially lethal target in ARID1A-mutant tumors, where depletion of ARID1B selectively impaired proliferation and caused destabilization of the SWI/SNF complex^3,4^.

Various publications have emphasized the importance of chromatin structure for DNA damage repair, particularly highlighting the process of chromatin remodeling required to facilitate access of repair enzymes to DNA ends^5^. As a key member of the chromatin remodeling family, increasing significance is attributed to the SWI/SNF complex in the processing of double strand breaks (DSB) especially during homologous recombination repair (HRR)^6^. Additionally to its involvement in HRR, the SWI/SNF complex was also linked to the recruitment of several factors involved in non-homologous end joining (NHEJ)^7^. Targeting of chromatin remodeling, particularly of the SWI/SNF complex^8^, may therefore prove to be a successful strategy for the sensitization of cancer cells to DNA-damaging therapy such as irradiation.

Here, we investigated the effect of ARID1B knockdown on radiosensitivity in colorectal cancer (CRC) cell lines by measuring clonogenic survival after irradiation of cells with and without ARID1A mutation. Additionally, formation of DSB repair foci such as RAD51 and 53BP1 was evaluated to determine the mechanism of radiosensitization in ARID1A-mutant CRC cells.

## Results

### ARID1B knockdown and its effect on radiosensitivity

**Figure 1:**
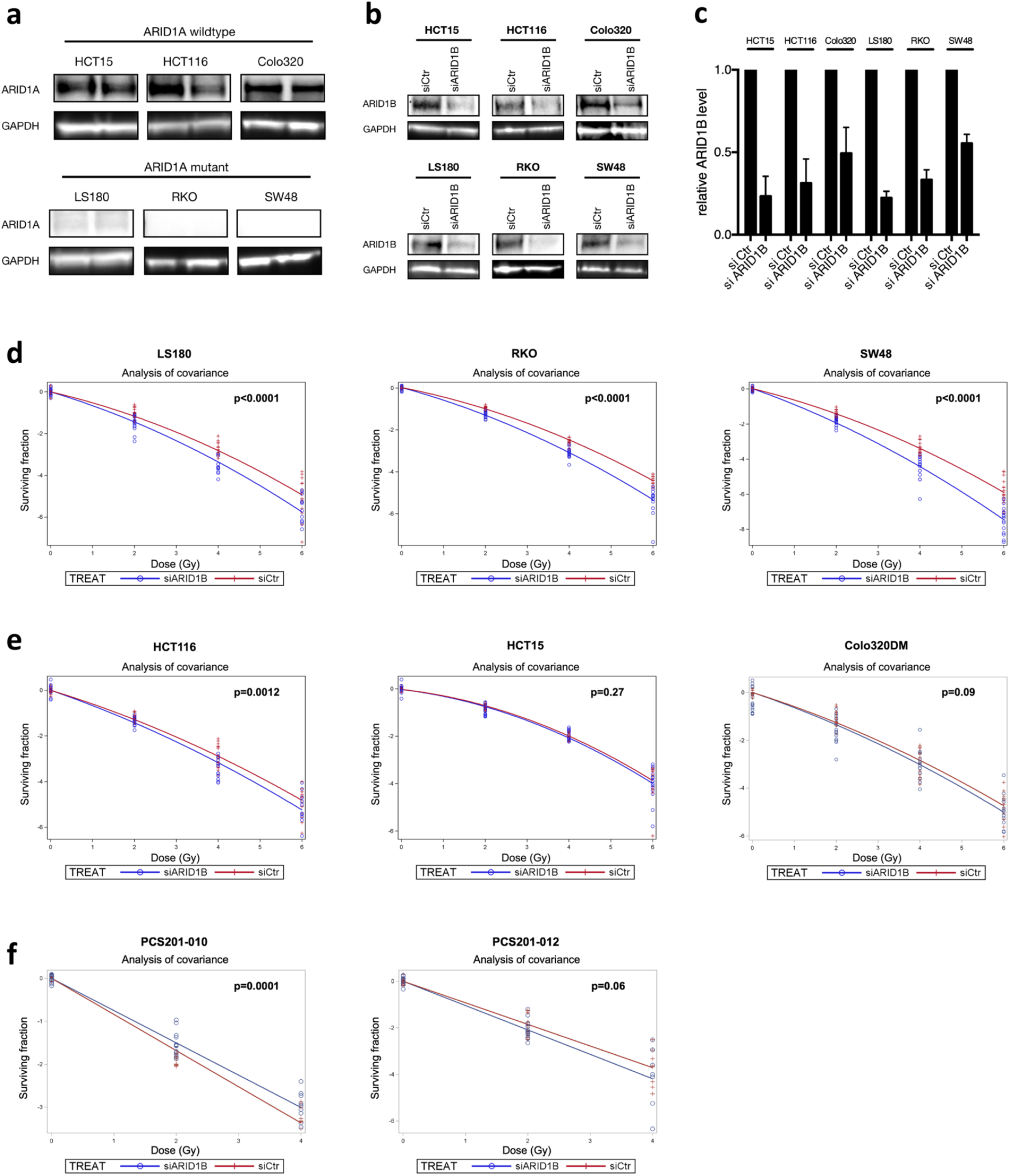
Effect of ARID1B knockdown on radiosensitivity. (**a**) ARID1A expression in wildtype and mutant cell lines was validated by western blot. (**b**) Representative immunoblots of ARID1B expression 48 h after transfection with siRNA targeting ARID1B and a nontargeting siRNA (siCtr) as control. Multiple blots acquired at similar exposures were combined and cropped to show relevant regions. GAPDH is shown as internal control. (**c**) Relative protein expression levels. Depletion of ARID1B sensitizes ARID1A-deficient cells to irradiation (**d**) but not ARID1A-proficient cells. (**e**) Depletion of ARID1B does not significantly sensitize fibroblast cell lines to irradiation, but decreases the slope of the radiation dose-dependent survival curve in PCS201-010. (**f**) Survival curves for ARID1B depleted and non-targeted siControl-transfected cells are shown together with residual values of the observed surviving fractions. Surviving fraction: Natural logarithm of the surviving fraction normalized to the mean of the sham irradiated controls, exposed to control- or ARIDIB-siRNA. P values indicate the results from the ANOVA F-test for the radiation response-modifying effect of ARID1B-knockdown. Results of 5 independent experiments are shown for CRC cell lines and of n = 3–4 for fibroblast cells.

Colorectal carcinoma cell lines with wild type (HCT15, HCT116, Colo320DM) and mutant ARID1A (LS180, RKO, SW48) were investigated. As shown in Fig. 1a, immunoblotting of ARID1A confirmed the mutation status of the CRC cell lines investigated in the present study. Mutations lead to loss of the full-length ARID1A protein, as also previously reported by Mouradov *et al*.^9^. Treatment with ARID1B targeted siRNA reduced the expression levels to about 26%, 38%, 50%, 22%, 37% and 55% in HCT15, HCT116, Colo320DM, LS180, RKO, and SW48, respectively (Fig. 1b,c).

To evaluate the impact ARID1B knockdown has on radiation sensitivity, colony formation assays were performed in ARID1A-proficient and ARID1A-deficient CRC cell lines. ARID1B knockdown lead to a significant reduction of the surviving fraction in CRC cells harboring ARID1A mutations (Fig. 1d). Radiation sensitivity of all ARID1A-deficient cell lines was increased over radiation dose levels of 2–6 Gy as compared to control. The dose modifying factors by ARID1B knockdown are shown in Table 1. These dose-modifying factors correspond to a decrease in the survival fraction to 87.3% ± 2.1%, 86.0% ± 1.1% and 77.2 ± 1.5% per 1 Gy in the dose range of 0–6 Gy for the ARID1A-mutant colorectal cell lines LS180, RKO and SW48, respectively (p < 0.0001, F-test). In comparison, no significantly increased radiation sensitivity could be observed in ARID1A-proficient cell lines HCT15, Colo320 and primary human adult dermal fibroblasts (PCS201-010, PCS201-012) (Fig. 1e,f). However, a small but significant sensitizing effect by ARID1B knockdown was observed in HCT116 with a dose-modifying factor of about half that observed in ARIAD1A mutated lines. The dose-modifying factors due to ARID1B knockdown were significantly smaller in ARID1A-deficient than in ARID1A-wildtype cells (Table 1a). The Spearman rank-order correlation between mutational status and dose-modifying factor reveals a significant correlation of ARID1A mutation and radiosensitizing effect of ARID1B knockdown (rs = 0.88, p = 0.02). Radiosensitivity of all CRC cell lines after control transfection is given in Table 1b at the endpoint of the surviving fraction at 4 Gy (SF4) in comparison to unirradiated controls. ARID1A mutated and non-muted cell lines had similar SF4 values.

**Table 1:** Radiosensitizing effect of ARID1B knockdown for ARID1A-deficient  and - proficient cell lines from the clonogenic assay.

Cell line	Dose modifying factor (α_ARID1B_ − α_ctr_) [Gy^−1^]	p
**(a)**		
SW48 (mt)	−0.259 ± 0.020	<0.0001
RKO (mt)	−0.151 ± 0.013	<0.0001
LS180 (mt)	−0.136 ± 0.024	<0.0001
HCT116 (wt)	−0.070 ± 0.021	0.0012
Colo320DM (wt)	−0.045 ± 0.026	0.092
HCT15 (wt)	−0.020 ± 0.019	0.27
PCS201-010	+0.090 ± 0.022	0.0001
PCS201-012	−0.120 ± 0.063	0.06
**(b) Surviving fraction at 4** **Gy of ARID1B-defiicent and - proficient cell lines from the clonogenic assay**		
**Cell line**	**Surviving fraction at 4** **Gy**	
SW48 (mt)	1.92% (95% CI: 1.49–2.48%)	
RKO (mt)	5.84% (95% CI: 5.15–6.62%)	
LS180 (mt)	4.27% (95% CI: 3.72–4.92%)	
HCT116 (wt)	4.43% (95% CI: 3.83–5.12%)	
Colo320DM (wt)	5.28% (95% CI: 4.39–6.35%)	
HCT15 (wt)	14.2% (95% CI: 13.2–15.4%)	
PCS201-10	4.55% (95% CI: 4.15–5.00%)	
PCS201-012	1.96% (95% CI: 1.38–4.08%)	

### Depletion of ARID1B inhibits initial RAD51 foci formation in ARID1A-deficient cells

**Figure 2:**
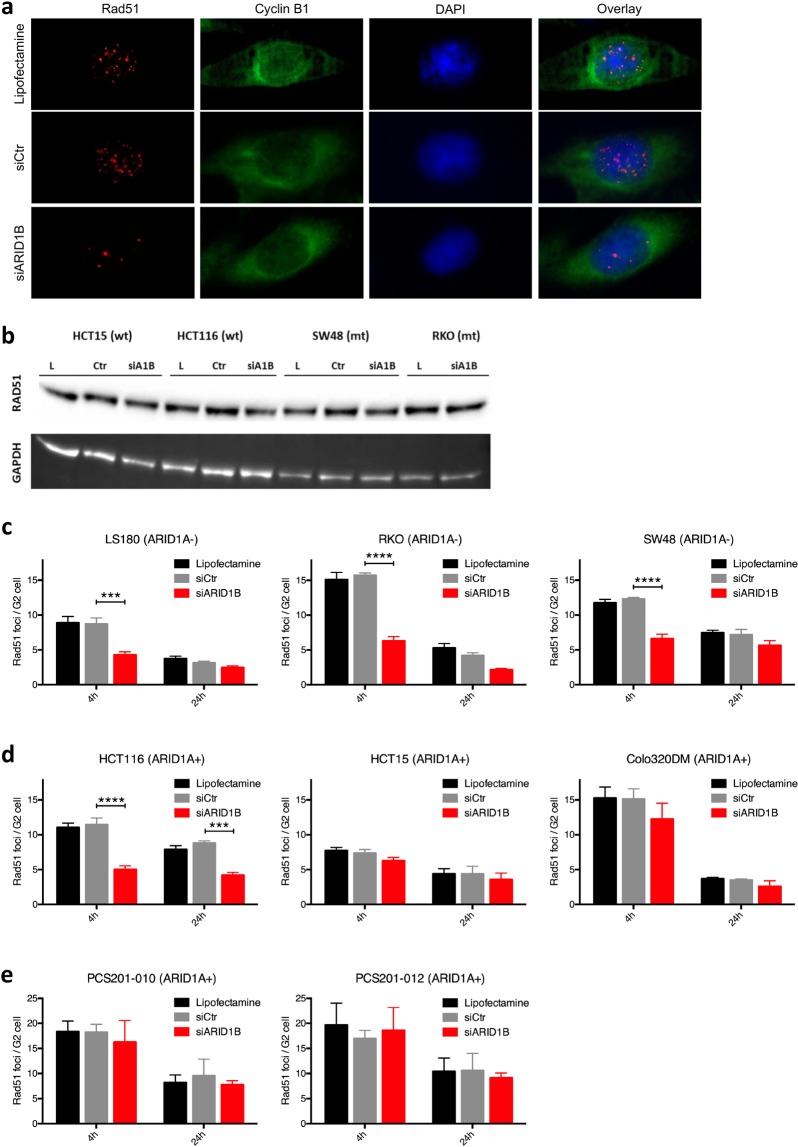
Effect of ARID1B knockdown on DSB repair signaling. (**a**) Representative immunofluorescent pictures of RAD51 foci formation in RKO cells (ARID1A mt) 4 hours after irradiation with 4 Gy. (**b**) RAD51 protein expression after transfection with lipofectamine (L), control siRNA (Ctr) and siARID1B (siA1B). The gel was cropped to show relevant information. Initial and residual Rad51 foci formation, as a measure for homologous recombination, in ARID1A-deficient (**c**) and ARID1A-proficient CRC cell lines (**d**) as well as fibroblast cell lines. (**e**) Cells were transfected for 48 hours with the indicated siRNA and then seeded for repair experiments, irradiated 4 hours later with 0 and 4 Gy, and fixed at the indicated time points of 4 and 24 hours after irradiation. Foci were analyzed in G2-phase cells after immunostaining for Rad51 as a marker for HRR, and cyclin B1 as a marker for G2 phase cells. Mean values with SEM are shown for at least 3 independent experiments, with asterisks indicating significant differences between siCtr and siARID1B as determined by the two-way ANOVA (***p < 0,001, ****p < 0,0001).

To elucidate the mechanistic background of radiosensitization in ARID1A-mutated cell lines, RAD51 foci formation as a marker of HRR was measured. Following ARID1B knockdown, cells were irradiated with 0 and 4 Gy and stained at 4 and 24 hours after irradiation. RAD51 foci formation was studied in cells counterstained with cyclin B1 as a marker for G2-phase cells, to only measure cells proficient in HRR as a pathway for DNA damage repair (Fig. 2a). Depletion of ARID1B significantly reduced the number of RAD51 foci at 4 hours (maximum of initial level) after irradiation in all ARID1A-mutated cell lines, as well as in the ARID1A-proficient cell line HCT116 (Fig. 2c), without an effect on RAD51 protein expression (Fig. 2b). RAD51 foci formation in the other investigated ARID1A-proficient cell lines HCT15 and Colo320DM was not affected (Fig. 2d). Similarly, AIRD1B knockdown did not affect RAD51 foci formation in the human fibroblast cell lines PCS201-010 und PCS201-012 (Fig. 2e).

**Figure 3:**
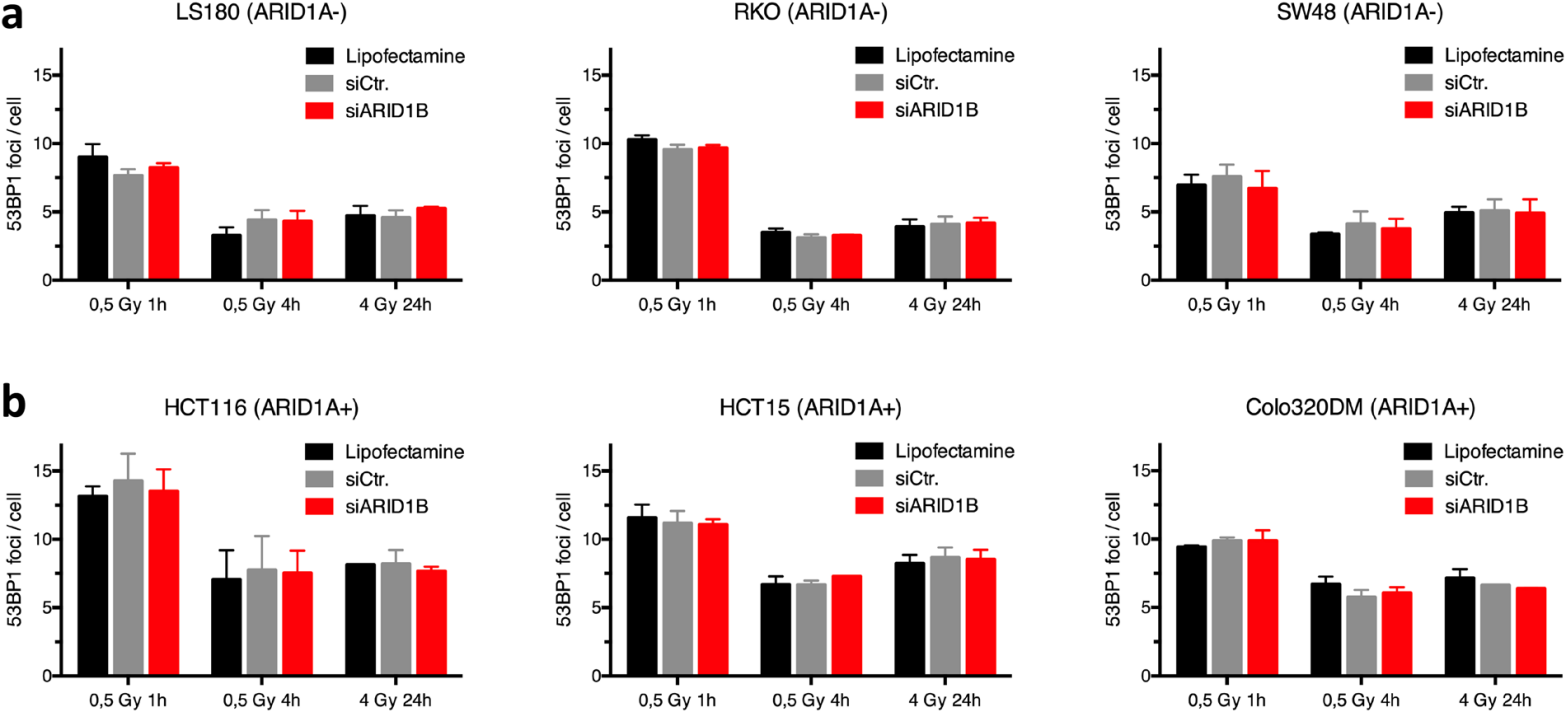
Effect of ARID1B knockdown on 53BP1 foci formation. As a measure of NHEJ, 53BP1 foci formation was analyzed at 1 h (initial) and 24 h (residual) after irradiation of ARID1A-deficient (**a**) and ARID1A-proficient CRC cell lines. (**b**) Following transfection, cells were seeded, irradiated 4 hours later with 0, 0.5 and 4 Gy, and stained at indicated time points after IR. Foci were analyzed after immunostaining for 53BP1 as a marker for NHEJ. Cells were transfected for 48 hours, harvested and fixated. Nuclei were stained with DAPI. Mean values are shown with standard deviation from 2–3 independent experiments.

**Figure 4:**
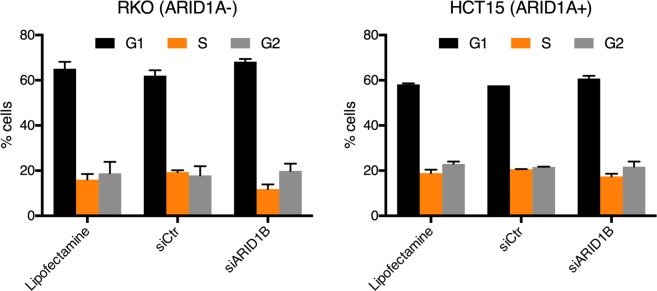
Effect of ARID1B knockdown on cell cycle progression. Cell cycle distribution after knock down of ARID1B exemplarily in ARID1A deficient (RKO) and proficient (HCT15) cell lines. Cells were transfected for 48 hours, harvested and fixated. Nuclei were stained with DAPI. Mean values are shown with standard deviation from at least three independent experiments.

Additionally, we investigated 53BP1 foci formation as a marker of non-homologous end-joining (NHEJ) activity following DNA damage. ARID1B knockdown did not affect 53BP1 foci formation in either the ARID1A-mutated or the ARID1A-wildtype cell lines at 1, 4 and 24 hours following irradiation with 0, 0.5 and 4 Gy (Fig. 3a,b). Furthermore, to exclude changes in cell cycle distribution following depletion of ARID1B as a reason for radiosensitization in ARID1B-mutated cell lines, we measured cell cycle distribution after transfection with siARID1B or siControl exemplarily in RKO and HCT15. Cell cycle profiles did not reveal significant changes following ARID1B knockdown neither in ARID1A-proficient (RKO) and –deficient (HCT15) cell lines (Fig. 4).

### Depletion of ARID1B reduces proliferation and plating efficiency in ARID1A-proficient CRC cell lines

**Figure 5:**
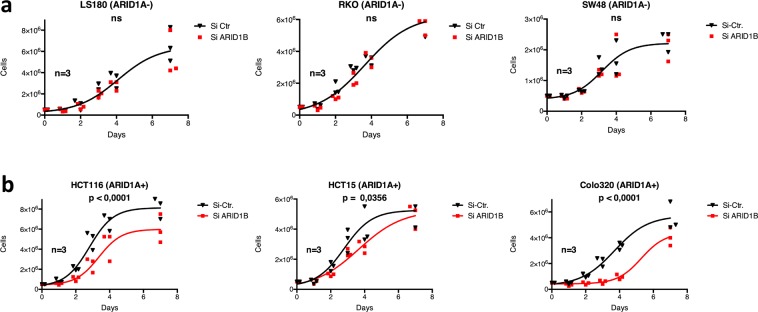
Effect of ARID1B knockdown on cell proliferation in ARID1A mutant (**a**) and wildtype cell lines (**b**). Proliferation of cells treated with siARID1B or control. At day 0, 0.5 × 10^6^ cells were seeded per flask (25 cm^2^), counted at day 1, 2, 3, 4, 7 and normalized to day 1. Results from 3 independent experiments are shown, with p values indicating significance between siCtr and siARID1B as determined by the F test on nonlinear regression.

To evaluate the effect of ARID1B knockdown on proliferation, we compared the short-term proliferative potential of ARID1A-proficient and ARID1A-deficient cell lines after depletion of ARID1B. Knockdown of ARID1B significantly inhibited proliferation of ARID1A-proficient, but not in ARID1A-deficient cell lines (Fig. 5). Colony formation assays were performed in ARID1A-proficient and ARID1A-deficient cells to evaluate the impact of ARID1B depletion on long-term survival. Downregulation of ARID1B led to a significant decrease in plating efficiency in all cell lines except LS180, the most pronounced effect being observed in the ARID1A-proficient cell lines Colo320DM and HCT116 (Table 2). The results from the short-term proliferation assay and the clonogenic survival assay reveal a significant inhibition of proliferation and clonogenic ability after ARID1B knockdown especially in ARID1A-proficient CRC cell lines, but less in ARID1A-deficient cell lines.

**Table 2:** Plating efficiencies of ARID1B-defiicent and –proficient cell lines from the clonogenic assay.

Cell line	ARID1A status	Median PE_ctr_ (interquartilerange)	Median PE_ARID1B_ (interquartilerange)	Median PE_ARID1B_/PE_ctr_ (interquartilerange)
SW48	mt	0.410 (0.390–0.465)	0.312 (0.260–0.370)	0.785 (0.592–0.880)
LS180	mt	0.180 (0.155–0.225)	0.195 (0.185–0.230)	1.000 (0.851–1.133)
RKO	mt	0.600 (0.555–0.615)	0.335 (0.270–0.405)	0.640 (0.432–0.711)
HCT15	wt	0.415 (0.365–0.460)	0.280 (0.180–0.305)	0.636 (0.432–0.789)
HCT116	wt	0.395 (0.305–0.500)	0.095 (0.060–0.205)	0.203 (0.167–0.432)
Colo320DM	wt	0.265 (0.195–0.335)	0.040 (0.010–0.099)	0.171 (0.069–0.318)
PCS201-010	wt	0.110 (0.095–0.145)	0.026 (0.014–0.034)	0.210 (0.134–0.260)
PCS201-012	wt	0.060 (0.027–0.093)	0.015 (0.010–0.019)	0.260 (0.161–0.405)

## Discussion

Identifying new cancer-specific vulnerabilities arising in the context of mutations within the SWI/SNF chromatin remodeling complex can be of pronounced clinical significance, and can potentially give rise to more effective use of radiotherapy through combination with chromatin-targeted therapy. In the present study, ARID1B knockdown in the context of ARID1A deficiency was evaluated for the effects on the radiosensitivity of CRC cell lines.

Our results demonstrate a novel approach to selectively increase radiation sensitivity in ARID1A-mutated cancers. The data from the colony formation assay reveal that ARID1B knockdown sensitizes ARID1A-deficient cells without considerably affecting proliferation or plating efficiency, highlighting the specific effect on radiosensitivity. In comparison, downregulation of ARID1B showed no sensitizing effect in the clonogenic assay for fibroblast cell lines and CRC lines with wild-type ARID1A, except for a minor sensitization of HCT116. While alterations in SWI/SNF composition have wide-spread roles in tumor biology, these findings describe a very confined and specific vulnerability of the residual SWI/SNF complex in cancers containing inactivating ARID1A mutations.

In order to further investigate the mechanisms of radiosensitization, we investigated whether ARID1B knockdown affects formation of DSB repair foci. Consistently with the data from the clonogenic survival assay, depletion of ARID1B had a significant impact on the radiation induced RAD51 foci formation in ARID1A-mutated cell lines. RAD51 foci formation in ARID1A-proficient colorectal carcinoma (CRC) cell lines was not affected, except for HTC116. In addition, siARID1B had no effect on RAD51 foci in two different normal skin fibroblast cells. Rad51 foci were measured in cyclin B1-positive cells alone, since HRR is present in S/G2 phase cells only, further refining this assay to specifically represent HRR activity. An altered interaction between members of the SWI/SNF complex and HRR enzymes following depletion of ARID1B might ultimately contribute to an increased radiosensitivity in ARID1A-mutated cell lines. However, much of these possible interactions between SWI/SNF subunits and DSB repair enzymes remain elusive. ARID1A is recruited to double strand breaks mediated by ATR^10^. In addition, ARID1B also accumulates at DNA-double strand breaks. Suppression of ARID1A or ARID1B could lead to reduced non-homologous end-joining^4^. While we did not find an effect of ARID1B knockdown on 53BP1 foci formation, a marker of NHEJ, in any of the CRC cell line, this does not rule out a role of NEHJ on the observed radiosensitizing effect of ARID1B knock down in ARID1A mutated CRC cell lines. Park *et al*. pointed out that in ARID1A deficient tumors, decreased accessibility of 53BP1 to DNA lesions leads to reduced NHEJ activity^11^. Comparing ARID1A -proficient and -deficient cell lines, we found a broad overlap in the surviving fraction at 4 Gy as a measure for radiosensitivity. Taken together, evidence exists from different studies that both NHEJ and HR can be affected by ARID1B knockdown. In ARID1A mutated cell lines, ARID1B knockdown led to a marked dose dependent radiosensitization, that was not dependent on an anti-proliferative effect. Growth inhibition or reduction in platting efficiency was minor or not present in these cell lines and this highlights the selectivity of the radiosensitizing effect. This radiosensitizing effect on the surviving fraction has the potential to be raised to high power during fractionated irradiation of colorectal carcinomas. In ARID1A-proficient CRC cells, as well as in fibroblasts, depletion of ARID1B led to a significant inhibition of proliferation without DNA damage, but not to a dose dependent radiosensitization. However, several of radiation dose-limiting normal tissues around a tumor are non-proliferating. Furthermore, the anti-proliferative effect of ARID1B knockdown in ARID1A proficient cells is consistent with previous findings describing opposing roles of ARID1A and ARID1B in tumor biology^12^. Depletion of ARID1B may result in an overbalance of the tumor suppressing properties of ARID1A within the SWI/SNF complexes, resulting in decreased proliferation of un-irradiated cells with wild-type ARID1A. Consistent with our study, Wang and colleagues demonstrated that ARID1B depletion inhibits proliferation in bladder cancer cell lines without ARID1A mutation^13^. Additionally, high levels of ARID1B expression have been linked to poor clinical outcomes in bladder urothelial carcinoma and breast carcinoma, while low ARID1A levels have been linked to poor clinical outcomes^13–17^. On the other hand, Helming and colleagues found a synthetic lethal relationship of ARID1A and ARID1B in a subgroup but not in all of the analyzed ARID1A mutated cancer cell lines, describing ARID1B as a specific vulnerability in ARID1A-mutant cancers^3^.

Here, we showed that independent from growth inhibition ARID1B knockdown can sensitize ARID1A mutated CRC cells to ionizing irradiation paralleled by a reduction of RAD51 foci induction indicating reduced homologous recombination. Effective treatment with small molecular inhibitors targeting EZH2, ATR and PARP has already been demonstrated in ARID1A-deficient tumors^10,18,19^. ARID1B is potentially drugable through its E3 ubiquitin ligase interaction^20^, and therefore similar approaches may be used in targeting ARID1B.

In conclusion, our study shows that radiosensitivity in ARID1A mutant CRC cell lines can selectively be increased through depletion of ARID1B, suggesting ARID1B as a potential therapeutic target to increase radiosensitivity in ARID1A-deficient tumors.

## Methods

### Cell lines

The human CRC cell lines LS180, RKO, SW48, HCT15, HCT116 and Colo320DM were obtained from ATCC (LGC Standards, Wesel, Germany). LS180, RKO and SW48 were kept in MEM (Invitrogen) supplemented with 15% FBS, 1% essential amino acids and antibiotics. HCT15, HCT116 and Colo320DM were kept in RPMI (Invitrogen) supplemented with 10% FBS plus antibiotics. Adult dermal fibroblasts (HDFa, PCS-201-012) and neonatal fibroblasts (HDFn, PCS-201-010) were obtained from ATCC. Fibroblasts were cultured with fibroblast basal medium (FBM, PCS-201-030 from ATCC) supplemented with low serum fibroblast growth kit (ATCC, PCS-201-041). Cells were cultivated at 37 °C in 5% CO_2_. Irradiation was done using the RS320 X-Ray machine by XStrahl Ltd. at 300 kV, 10 mA, dose rate 0,9 Gy/min.

### Transfection with siRNA

Cells were seeded in 6-well plates and incubated for 24 hours, obtaining a 70–80% confluent monolayer. Cells were then washed in HBSS and OptiMEM (both Gibco), and subsequently incubated with transfection reagent for 4 hours. We used 500 µl OptiMEM with 80 nM siRNA and 6 µl Lipofectamine RNAiMAX (Invitrogen) for transfection reagent. To downregulate ARID1B, we established a mix of two siRNAs (Ambion s199170, Ambion s199168,) at 40 nM each for optimal efficiency. As controls, we used non-targeting siRNA (Ambion 4390843) as well as H_2_O. After 4 hours of incubation with the transfection reagent, 500 µl of culture medium with double FBS was added and cells incubated for 48 hours until harvesting for further experiments. Expression of targeted proteins was regularly checked by Western blots.

### Immunoblotting

Western blots were performed with anti-ARID1A (CellSignalling, 12354P), anti-ARID1B (LS-Bio, LS-C382223), anti-RAD51 (Calbiochem, PC130-100UL) and anti-GAPDH (Abcam, ab8245). The secondary antibodies were HRP-linked antibodies raised against mouse or rabbit IgG (GE Healthcare, NA931V and NA934V) and Alexa Fluor 488-linked antibodies against mouse (Invitrogen A11029).

### Cell proliferation assay

48 hours after transfection, 0,5 × 10^6^ cells were seeded per 25 cm^2^ flask. Cells were harvested and counted by automatic counting system Luna (Logos Biosystems) at day 1, 2, 3, 4, 7 after seeding.

### Clonogenic survival assay

48 hours after transfection, cells were harvested and plated in triplicates in 9,6 cm^2^ culture dishes. After 4–6 hours in culture, cells were irradiated and subsequently incubated for 10–14 days at 37 °C in 5% CO_2_. Cells were fixed and stained with 96% ethanol, 15% Giemsa and destained with distilled water. Colonies consisting of at least 50 cells were counted. Surviving fractions after the indicated treatments are presented as a fraction of the growth of non-irradiated colonies^8^.

### Immunofluorescence analysis

48 hours after siRNA transfection, cells were harvested, reseeded in chamber slides and irradiated 4 hours later. At indicated times after IR, cells were washed, fixed, permeabilized and incubated with blocking buffer. Primary antibodies anti-RAD51 (Calbiochem, PC130-100UL), anti-cyclin B1 (EMD Millipore, 05-373) and anti-53BP1 (Abcam, ab21083) were added to the cells and incubated overnight. Secondary antibodies anti-mouse Alexa Fluor 488 (Invitrogen, A11017) and anti-rabbit Cyanine Cy3 (Jackson, 111-165-008) were applied together with 1 µg/ml DAPI (Invitrogen) nuclear counterstain for 1,5 hours at room temperature. Slides were examined on the fluorescent microscope imager Z1 (Zeiss).

### Statistical analysis

Colony data were analyzed using a linear-quadratic model describing the dependence of the logarithm of cellular survival on dose. The interaction between ARID1B knock down and the radiation dose response was described as a slope modifying effect of the linear term of the linear-quadratic model (Procedure GLM, SAS/STAT 14.1, SAS Institute Inc. Version, Cary, NC, USA). Higher order effects were considered if significant at alpha = 0.01.

## Data Availability

All relevant data not presenting in the main figures is available from the authors.
